# From Endogenous to Synthetic microRNA-Mediated Regulatory Circuits: An Overview

**DOI:** 10.3390/cells8121540

**Published:** 2019-11-29

**Authors:** Elsi Ferro, Chiara Enrico Bena, Silvia Grigolon, Carla Bosia

**Affiliations:** 1 IIGM—Italian Institute for Genomic Medicine, c/o IRCCS, 10060 Candiolo (Torino), Italy; 2 Candiolo Cancer Institute, FPO-IRCCS, 10060 Candiolo (Torino), Italy; 3 The Francis Crick Institute, 1 Midland Road, London NW1 1AT, UK; 4 Department of Applied Science and Technology, Politecnico di Torino, Corso Duca degli Abruzzi 24, 10129 Torino, Italy

**Keywords:** microRNA, network motifs, synthetic circuits

## Abstract

MicroRNAs are short non-coding RNAs that are evolutionarily conserved and are pivotal post-transcriptional mediators of gene regulation. Together with transcription factors and epigenetic regulators, they form a highly interconnected network whose building blocks can be classified depending on the number of molecular species involved and the type of interactions amongst them. Depending on their topology, these molecular circuits may carry out specific functions that years of studies have related to the processing of gene expression noise. In this review, we first present the different over-represented network motifs involving microRNAs and their specific role in implementing relevant biological functions, reviewing both theoretical and experimental studies. We then illustrate the recent advances in synthetic biology, such as the construction of artificially synthesised circuits, which provide a controlled tool to test experimentally the possible microRNA regulatory tasks and constitute a starting point for clinical applications.

## 1. Introduction

Life as we see it is the result of the complex regulation of organisms’ gene expression, required to fulfil vital biological tasks. This complexity is well reflected by the intricate network of interactions between the thousands of genes constituting genomes. Specifically, these interactions are triggered by the action of transcription factors (TFs), which are the regulatory molecules carrying out the primary step of either enhancing or inhibiting their target gene’s transcription. The sum of all transcriptional regulatory relationships throughout an organism’s genome constitutes its transcription network.

Originally, transcription networks were found to be built up of recurring patterns, called network motifs [1]. Identical motifs have been observed in different organisms ranging from bacteria to humans, indicating that they constitute the basic subcomponents of transcription networks [2]. The most acknowledged small scale motifs involve single or multiple target genes and one or more TFs as master regulators. Depending on its topology, each type of circuit can carry out defined information processing functions that allow achieving a specific biological task [3]. For example, positive self-loops slow down dynamical responses and increase variability, whereas negative auto-regulation is well known for accelerating responses and decreasing noise [4,5]. Coherent feedforward loops can display response persistence, while incoherent feedforward loops show a hallmark pulse-like outcome and speed up response. Single input modules, made up of a single TF regulating multiple genes, can generate temporal expression programmes [2].

The same motifs were later found in mixed regulatory networks, that is in networks that are not necessarily limited to transcriptional interactions [6,7], and multiple studies have highlighted the role of microRNAs (miRNAs) as pivotal players in these circuits [8].

MiRNAs are evolutionarily conserved ∼22 nt long non-coding RNA molecules that act as post-transcriptional gene regulators. Such regulation is achieved through Watson–Crick base pairing with the target mRNAs, mainly at their 5′ terminus or inside their 3′-UTR region [9]. The strength of binding, that is the degree of miRNA–mRNA complementarity, determines whether the target mRNA will be subject to translational repression or mRNA degradation: partial complementarity inhibits translation, while near-perfect matching results in its degradation [10,11]. Although a large number of miRNA genes are dispersed over the genome in intergenic regions, more than half of miRNAs reside in introns of their host genes [12], and bioinformatic analyses suggest that they are usually oriented on the same DNA strand of the host [13]. Thus, miRNA biogenesis occurs from both independently transcribed and intron-embedded primary miRNAs [14].

The minimum requirement for miRNA–mRNA interaction is a binding sequence of six consecutive nucleotides, often called “miRNA seed” [15]. Such a soft constraint allows an miRNA molecule to bind potentially hundreds of different genes [10]. Indeed, miRNAs typically act in a combinatorial fashion: a single gene is often targeted by many different miRNAs, and a single miRNA regulates multiple genes [16]. So far, over 2500 different mature miRNAs have been observed in humans [11], and an increasing number of regulatory interactions involving miRNAs is currently being uncovered both by theoretical prediction methods and experimental validation [17].

The way miRNAs interact with their targets is titrative [18]. From a theoretical point of view, titration mechanisms are characterised by a threshold effect close to the equimolarity of the different interacting species, hypersensitivity of the system around the threshold and cross-talk among targets [19,20,21]. The latter characteristic, also identified as a competing endogenous RNA (ceRNA) effect, marks indirect interactions among targets in competition for a common pool of miRNAs [22,23]. These targets may include protein coding messenger RNAs and non-coding RNAs such as long non-coding RNAs [24,25], pseudogenes [26,27] and circular RNAs [28] that act as natural miRNA sponges, thus co-regulating each other in an miRNA mediated fashion. Within this context, the notion of trios comprised of one miRNA, one non-coding RNA sponging the miRNA, and one target messenger RNA has received increasing attention.

MiRNAs regulate fundamental biological processes, such as embryonic development and synaptic plasticity [6], and play pivotal roles in disease related contexts including diabetes, viral infection [29] and tumourigenesis [30,31]. Along with the interaction of miRNAs with TFs in the most diverse biological contexts, their relationship with epigenetics is being increasingly uncovered [32]. However, it is still partially unclear why miRNAs are so pervasive and conserved. Although there are examples of in vivo transgenic models either overexpressing or silencing specific miRNAs that produce a phenotype [33,34,35,36], single miRNAs generally exert weak repressive action on their target genes and rarely show phenotypic outcomes [37].

Furthermore, the results obtained by analysing multi-omics data obtained by over- or down-expressing a single miRNA in a given cell-line are not always in line with this idea. For instance, M’baya-Moutoula and collaborators studied the effects of both enhancement and inhibition of a metabolic disease related miRNA, *miR-223*, in a monocyte macrophage cell-line [38]. After the identification of a great number of candidate regulators and targets of *miR-223*, the most significantly affected ones were uncovered to be key metabolites. Thus, *miR-223* alone seems to be able to clearly alter cells’ metabolic profiles, possibly impacting on apoptosis and proliferation. Furthermore, *miR-34a*, a notable tumour suppressor, has been identified as a hub in miRNA involving regulatory networks [39]. Specifically, Hart and colleagues [39] investigated its influence on genes regulating the immune system in three different T-cell-lines. Fourteen *miR-34a* targets out of 193 candidates were experimentally confirmed to affect T-cell function. Hence, the authors suggested a crucial role of *miR-34a* in T-cell regulation that could be exploited to modulate immune response in cancer related contexts for therapeutic purposes.

The identification of therapeutic miRNA targets has been lately addressed by Calsina and colleagues [40] with a multi-omics data analysis in the context of rare neuroendocrine tumours [40]. Prognostic miRNA markers for two specific types of tumour were searched for by integrating transcriptome, proteome and miRNome data and by validating predictions in vitro. Among the group of miRNAs identified as a signature of metastasis and progression, a combination of two of them displayed the strongest correlation with metastatic risk. Specifically, the *miR-21-3p/miR-183-5p* axis by itself appears to have the power to critically affect tumour development. Genes altered in the same cancer types were also linked to overexpression of the 182/96/183 miRNA cluster and to knockdown of the DLK1-MEG3 miRNA cluster [41].

In essence, even though the single miRNA–target interaction seldom displays phenotypic outcomes, a number of works suggest that single miRNAs or families involved in a broader interactome, where their alteration might give rise to cascade-like consequences, can concretely affect cell state and function.

The emerging idea is that miRNA mediated regulation could serve to manage the impact of noise in gene expression [42]. A number of studies have addressed the relationship between miRNA and noise, and the results suggested that miRNAs could provide precision to protein expression by buffering noise when needed [37,43] or either exploit stochasticity in biological contexts that take advantage of gene expression variability, such as differentiation processes [44,45].

In this review, we first focus on the roles of microRNAs in network motifs, which will be discriminated by their different topologies. We will then address artificially synthesised circuits, which provide additional insight into the possible regulatory tasks that miRNAs can potentially accomplish and ways of taking advantage of miRNAs’ functionalities in artificial systems.

## 2. MicroRNAs in Network Motifs

The interplay between miRNAs and TFs in a mixed regulatory circuit, where transcriptional, post-transcriptional and epigenomic regulatory interactions are joined together, has been proposed as a guiding structure for the achievement of genetic tasks. The influence of TFs and epigenetic regulators on miRNAs and vice versa was initially addressed computationally and also recently confirmed experimentally, mainly using RNA sequencing (RNA-seq) and CLIP (crosslinking followed by immunoprecipitation) sequencing (CLIP-seq). Analyses of data from the Encyclopedia of DNA Elements (ENCODE) showed that miRNA mediated circuits are found in real networks significantly more often than expected by chance, as reviewed in [17,46].

Along with the identification and experimental validation of over-represented network motifs, which has been possible thanks to the interplay between bioinformatics and computational methodologies, as well as high throughput and sophisticated modern technologies, mathematical modelling has been shown to be a powerful tool in the understanding of motif functionality. In the models formulated over the past years, the miRNA mediated gene expression regulation is studied as a dynamical system. More broadly, such modelling has also been used for investigating cell and tissue functions at both spatial and temporal resolution and used as a prediction tool for drug and therapy development [11]. As clearly discussed in [11], the key ingredients of the modelling of such gene regulatory networks are the molecular species that compose the network and their interactions. Gene expression is currently well understood to be a stochastic process [47]. Given the probabilistic nature of chemical reactions and the small number of molecules involved, fluctuations in the abundance of such molecules are relevant. Thus, through stochastic modelling, several works deeply investigated the properties and biological advantages of such networks in the presence of different sources of noise [45,48,49].

**Figure 1:**
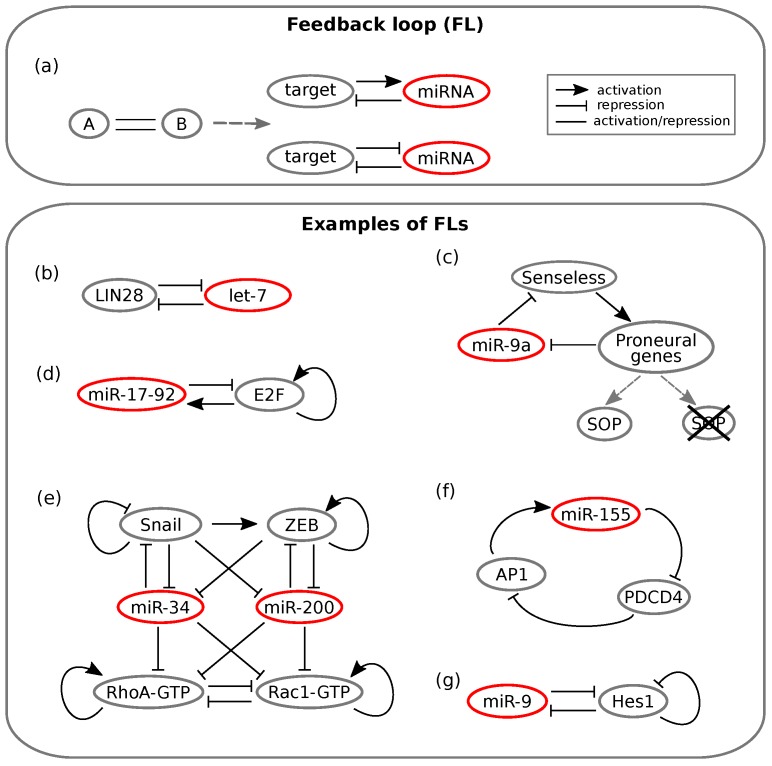
Schemes of feedback loops (FLs). Lines ending with an arrow indicate activation, whereas T ending lines indicate repression. Simple straight lines indicate that the interaction can either be activatory or repressive. (**a**) On the left, the general FL scheme is represented. On the right, the two possible configurations of a minimal miRNA mediated FL are reported. Adapted from [2]. (**b**–**f**) Experimental examples of miRNA mediated FLs. (**b**) Toggle switch between LIN28 and the *let-7* miRNA involved both in inflammation and non-small cell lung cancer. Adapted from [59]. (**c**) Double negative FL between Senseless and *miR-9a* that regulates *Drosophila* sense organ specification. Adapted from [42]. (**d**) Cancer associated positive FL between the *miR-17-92* family and E2F. E2F self-activation is also represented. Adapted from [60]. (**e**) Regulatory relationships that control transitions between epithelial, mesenchymal and amoeboid phenotypes. The network is composed of multiple feedback-like interactions. Adapted from [61]. (**f**) Positive feedback loop between *miR-155* and AP1 involved in tongue cancer progression. Adapted from [62]. (**g**) Toggle switch between *miR-9* and Hes1 that controls timing of neuron differentiation. Adapted from [63].

**Figure 2:**
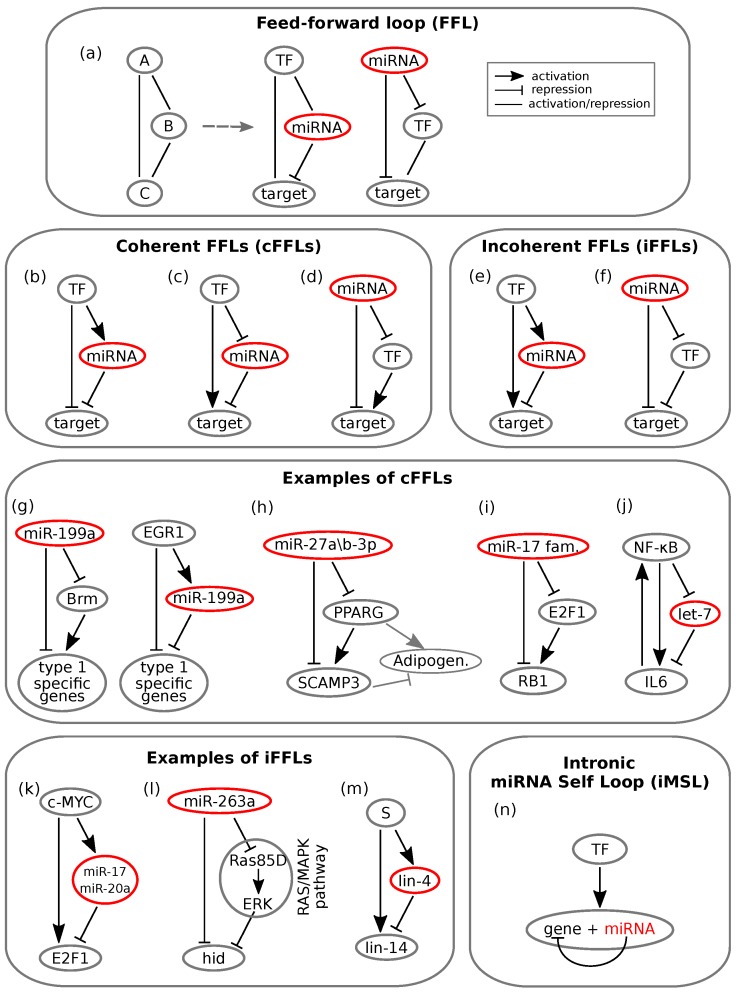
Schemes of feedforward loops (FFLs). Lines ending with an arrow indicate activation, whereas T ending lines indicate repression. Simple straight lines indicate that the interaction can either be activatory or repressive. (**a**) On the left, the general scheme of an FFL is represented. The master regulator gene *A* regulates gene *C* both directly (left path) and indirectly (right path) through the direct regulation of gene *C*. The central and right schemes synthesize all possible miRNA mediated FFLs: the miRNA can either play the role of a master regulator or standing in the intermediate regulator position. (**b**–**d**) Coherent miRNA mediated feedforward loops (cFFLs): regulatory paths make an overall positive sign. In (**b**) and (**c**), the TF plays the role of the master regulator, while in (**d**), such a role is assumed by the miRNA. (**e**,**f**) Incoherent miRNA mediated feed-forward loops (iFFLs): regulatory paths make an overall negative sign. In (**e**), the TF acts as the master regulator by activating both the miRNA and the target; in (**f**), the master regulator is the miRNA that represses both its targets. One of the targets then represses the other one. (**g**–**j**) Experimental examples of miRNA mediated cFFLs. (**g**) Two FFLs regulating the “all-or-none” expression of two groups of type 1 specific genes in human epithelial tumour cells. Adapted from [105]. (**h**) FFL mediated by *miR-27a/b-3p* regulating the expression of two target genes (PPARG and SCAMP3) in adipogenesis. The expression of PPARG upregulates SCAMP3. Adipogenesis has been found to be positively regulated by PPARG (grey arrow) and negatively regulated by PPARG (T-ended grey line). Adapted from [104]. (**i**) miRNA mediated FFL that fine tunes G0/G1-S transition during the cell cycle: the *miR-17* family regulates both E2F1 and RB1, whose expression inhibits E2F1. Adapted from [117]. (**j**) Oncogenic transformation is regulated by miRNA *let-7* through a cFFL: NF-κB regulates IL6 directly and indirectly through *let-7*. A feedback is present between IL6 and NF-κB. Adapted from [52]. (**k**–**m**) Experimental examples of miRNA mediated incoherent feed-forward loops (iFFLs). (**k**) iFFL that controls proliferative signal: the proto-oncogene *c-MYC* directly controls E2F1 transcription and indirectly controls its translation through activation of *miR-17* and *miR-20a*. Adapted from [118] (**l**) *miR-263a* controls cell crowding in the fly retina development, by regulating the pro-apoptotic gene *head involution defective* (hid) both directly and indirectly through the RAS/MAPK pathway. Adapted from [119]. (**m**) The miRNA *lin-4* dampens expression oscillations of its target miRNA *lin-14* through an FFL where both miRNAs are synchronously transcribed by a pulsatile source S. Adapted from [120]. (**n**) General scheme of an miRNA mediated intronic self-loop.

Specifically, a few miRNA mediated circuits are recurring network motifs in mixed regulatory circuits (Figure 1 and Figure 2). Tsang and colleagues [6] developed a computational method for analysing gene expression data, and the results indicated that two classes of circuits are enriched in the human genome, namely miRNA mediated feedback and feedforward loops (FFLs). More precisely, miRNAs appear to be often involved in negative feedback circuits and in both coherent and incoherent FFLs. Moreover, intronic miRNA mediated self-loops (iMSLs), where an miRNA is encoded within an intron of one of its targets, are also significantly represented in the human regulatory network [13], suggesting their potential to implement biologically relevant tasks. In this section, we will discuss the main properties of the most common miRNA mediated motifs, reviewing both theoretical and experimental studies. These will include miRNA mediated feedback and feed-forward loops, with double negative feedback loops and intronic miRNA mediated self-loops as peculiar cases.

### 2.1. MicroRNA Mediated Feedback Loops

Feedback loops (FLs) are among the over-represented miRNA motifs. An FL is a circuit whose topology is such that the output of the network influences the state of the input. This way, the output literally “feeds back” on the input. Positive and negative feedback regulation is intuitively useful. For instance, a TF can limit its own activity by activating a repressor, whereas a double negative feedback loop can function as a switch between cell states by producing a binary output [42].

As mentioned, an increasing number of TF-miRNA circuits have been identified as FLs [11]. In these circuits, as shown in Figure 1a, a TF either enhances or suppresses the expression of an miRNA, which in turn post-transcriptionally suppresses the TF [50]. It has been shown that this kind of circuit can implement a variety of biological functions, ranging from the conversion of a transient upstream signal in a long-lasting cellular response [6] to the achievement of bistable gene expression and buffering of noise, be it due to fluctuating transcription, translation and decay processes (intrinsic noise) or to external (e.g., environmental) factors (extrinsic noise) [11].

The first mentioned feature, that is the response persistence, has been observed for instance in human breast cells, where an FL involves the *let-7* miRNA [51]. The activation of the tyrosine-kinase Src triggers NF-κB, a TF known for inducing inflammatory response [52]. NF-κB in turn activates LIN28, while LIN28 inhibits *let-7* [52]. Since the latter represses IL-6, the consequence of a Src activation is a reduced suppression of IL-6 by the miRNA [52]. IL-6 completes the loop by activating NF-κB; the result is an FL that maintains the cell state triggered by Src activation also in the absence of the initial input [16]. However, the interaction between *let-7* and LIN28 appears to be more complex: it is proposed that they in turn form a double negative FL [53]; see Figure 1b. Recently, the *let-7*/LIN28 switch has been related to non-small cell lung cancer as well: Yin and colleagues showed that dysregulation of this circuit, which is induced by irradiation or chemotherapeutic drugs, can lead to radio- and chemo-resistance [54].

As a consequence of their importance in determining phenotypic outcomes, miRNAs appear to be largely involved in cancer related feedback motifs [55]. A well known example involves the E2F transcription factor, which is notoriously implicated in the regulation of cancer associated phenotypes. E2F can be regulated by multiple miRNAs belonging to the *miR-17-92* cluster [56]. The cluster is induced by E2F, while some miRNAs of the cluster inhibit E2F post-transcriptionally, thus forming an FL (see Figure 1d). Lai and co-workers [11] showed by ODE modelling and stochastic simulation that the miRNA cluster can either function as an oncogene or tumour suppressor depending on the values of two key parameters: the growth factor signalling intensity and the inhibition of E2F translation by the miRNA cluster. Moreover, they showed that introducing noise into external signalling or into E2F expression, the miRNA cluster functions as a noise buffer, thus conferring robustness to the network. However, there exist several other regulatory loops involving miRNAs in the E2F family interactome [57]; thus, the E2F regulation network will require further modelling efforts.

A newly published work by Zargar et al. [58] linked *miR-155* to Pdcd4 (programmed cell death 4) and AP-1 in a net positive feedback loop whose different outcomes can lead either to tongue cancer progression (*miR-155* overexpression) or to apoptosis and regression of tumour size (*miR-155* knockdown); see Figure 1f.

Along with their protein expression fine tuning and noise buffering properties, it appears that miRNA mediated FLs can generate oscillatory gene expression under some conditions [64]. In this type of regulatory pattern, the FL components are alternatively repressed or activated over time [11]. For instance, if a TF slowly activates the transcription of an miRNA, whereas the miRNA rapidly inhibits the TF by repressing its translation or enhancing its degradation, sustained oscillations emerge [11]. Xue and coworkers [65] examined another loop involving NF-κB: the indirect negative FL between *miR-21* and NF-κB. This circuit was shown to produce oscillations in NF-κB levels. On the other hand, the related negative FL formed by *miR-146ab* and IL-6 contributed by dampening the oscillations [11].

Oscillatory expression patterns play a fundamental role in the timing of neuron differentiation. Goodfellow et al. [63] studied the negative FL involving *miR-9* and Hes1 (Figure 1g), and they found out that *miR-9*, which is regulated by NF-κB [66], controls the oscillatory pattern of Hes1 expression. Again, the presence of an miRNA sets a threshold on the amount of a TF, Hes1: Hes1-high ES cells commit to the mesodermal fate, whereas Hes1-low ES cells are induced to differentiate as neuron stem cells [67]. Another work by Moore and colleagues [68] dealt with the p53 oscillations observed in breast cancer cells upon DNA damage induction. Their results suggested that this oscillatory behaviour is the outcome of a network of nested FLs where miRNAs tune the amplitude of oscillations [11].

The circadian clock represents a fundamental oscillatory gene expression program that adapts cell activity according to environmental fluctuations, most notably light changes [69]. MiRNA mediated regulation contributes crucially in controlling and maintaining circadian clocks in animals [70]. More specifically, multiple experimental studies on *Drosophila*, mouse and human cells [69,71,72,73] have found out that circadian rhythms are governed by several miRNA mediated FLs. Although mathematical modelling studies concerning these circuits are currently lacking, miRNAs may play important roles in conferring robustness to circadian rhythms by controlling noise and oscillations, similarly to what has been observed in other contexts [11].

The issue of how the composition of miRNA pools with a common target influences the target regulation is still under debate. Furthermore, it raises the question of how the variability of miRNAs’ abundances is achieved [44]. Interestingly, a newly published study [74] addresses the regulation of miRNA abundance during ageing, suggesting that it is controlled by an miRNA mediated FL. A global decline in miRNA amount with age has been shown, amongst others, in mice [75]. Inukai and coworkers [74] investigated the miRNA abundance decline in *C. elegans* and bioinformatically identified Alg-1/Argonaute as a regulator of global miRNA biogenesis. Alg-1 is putatively targeted by multiple miRNAs, yet experimental evidence showed that *miR-71* post-transcriptionally represses Alg-1, establishing an FL that influences global miRNA abundance during ageing and concomitantly increases mRNA expression variability [74].

Several further works could be brought forward to highlight the role of miRNAs in carrying out diverse biological functions through FLs. For additional references, see [60,62,76,77,78,79,80,81,82,83,84,85,86].

#### Double Negative Feedback Loops

The double negative feedback loop (DNFL), also known as toggle switch, is a minimal motif that allows “all-or-none” outcomes, which makes it a suitable processing system in cell fate decision [16]. This kind of switch has also been found as a recurrent motif in the epi-miRNA network [17]. Several examples of miRNAs directly interacting with epigenetic machinery have been uncovered as reciprocally inhibitory switches [7]. For instance, the *miR-29* family was shown to downregulate de novo DNA methylation by targeting two different methyltransferases. Interestingly, this miRNA cluster was hypermethylated itself in lung cancer, leading to overexpression of the same methyltransferases [87]. The involvement of many miRNAs in DNFLs, along with mathematical modelling and experimental proofs, supports the idea that miRNAs provide stability to alternative phenotypes in the presence of noise [88].

Since miRNAs play important roles in determining cell fate, their involvement in developmental contexts has been increasingly highlighted [89,90]. For instance, the *Drosophila* sense organ specification is achieved through an miRNA mediated DNFL [91]. Which cell will become part of the sense organ precursor (SOP) is determined stochastically, as it depends on the fluctuations in the level of the Senseless TF [42]. The DNFL linking *miR-9a* and Senseless (Figure 1c) is able to transform this noise into a switch: *miR-9a* levels are initially uniform among proneural cells, but as Senseless activity increases proneural gene expression in the SOP, *miR-9a* is inhibited, while it still remains high in the surrounding cells: spatially exclusive miRNA domains are achieved [42]. Thus, the presence of *miR-9a* ensures that only if Senseless levels overcome a threshold, cells commit to differentiation [42]. Indeed, the generation of mutant flies with extra sense organs has been observed in cases of *miR-9a* loss-of-function [92]. A bistable switch is found also in the development of the *C. elegans* chemosensory neurons [42]. These are meant to be asymmetric on the left and right sides of the animal: cells must adopt one of two alternative phenotypes, ASEL and ASER [93]. Their initial state is unstable, and thus subject to fluctuations by noisy gene expression: the *miR-273* family works together with TFs in a combination of loops that drives cells either to the left or the right type [42].

A DNFL between MALAT1 and *miR-663a* has recently been proposed as crucial in colon cancer cell functions [94], and the cancer associated fibroblast phenotype (CAF) has been shown to be tuned by *miR-145* in a reciprocally inhibitory regulatory motif [95]. Another notable cancer related case is that of two miRNA mediated DNFLs that regulate the switch between epithelial and mesenchymal phenotypes in metastasis [96]. The epithelial to mesenchymal transition (EMT) provides cells with the ability to migrate, thus initiating metastasis. The reverse transition (MET) allows colonisation and growth of metastases [96]. The two opposite transitions are controlled respectively by the *miR-34*/SNAIL and *miR-200*/ZEB DNFLs, which have been proposed to function together as a bistable switch [97] (see Figure 1e). Meanwhile, Lu and colleagues [59] showed that the two DNFLs have separate roles in the switch: ZEB/*miR-200* FL effectively carries out the EMT, whereas the SNAIL/*miR-34* loop functions as a noise buffer of external signals that could lead to aberrant EMT activation.

Model analysis showed the existence of a third hybrid phenotype with both migratory and adhesion properties that could enable collective cell migration [98]. Experimental observations are consistent with tristability, suggesting that medium levels of *miR-200* and ZEB lead to partial EMT [11]. Later, Huang and co-workers [61] connected the EMT network with the Rac1/Rhoa circuit that regulates transitions between the mesenchymal and the amoeboid phenotypes, as shown in Figure 1e. Model simulations showed that the transitions between individual and collective migration behaviours are regulated by *miR-200* and *miR-34*: high levels of the miRNAs favour individual migration, thus likely inhibiting the formation of cancer metastasis [11]. One of the most recent developments concerning the role of miRNAs in the epithelial-mesenchymal transition has been pursued by Diepenbruck and colleagues [99]. Among a number of different miRNAs, they identified *miR-1199-5p* as a strong regulator of EMT. Furthermore, the results showed that *miR-1199-5p* acts in a DNFL with the previously mentioned TF ZEB1, and the behaviour of the circuit seems similar to that of the *miR-200* family [99]. However, since *miR-200* and *miR-1199-5p* only share a few common target genes, they likely carry out distinct functions during the transition [99].

**Figure 3:**
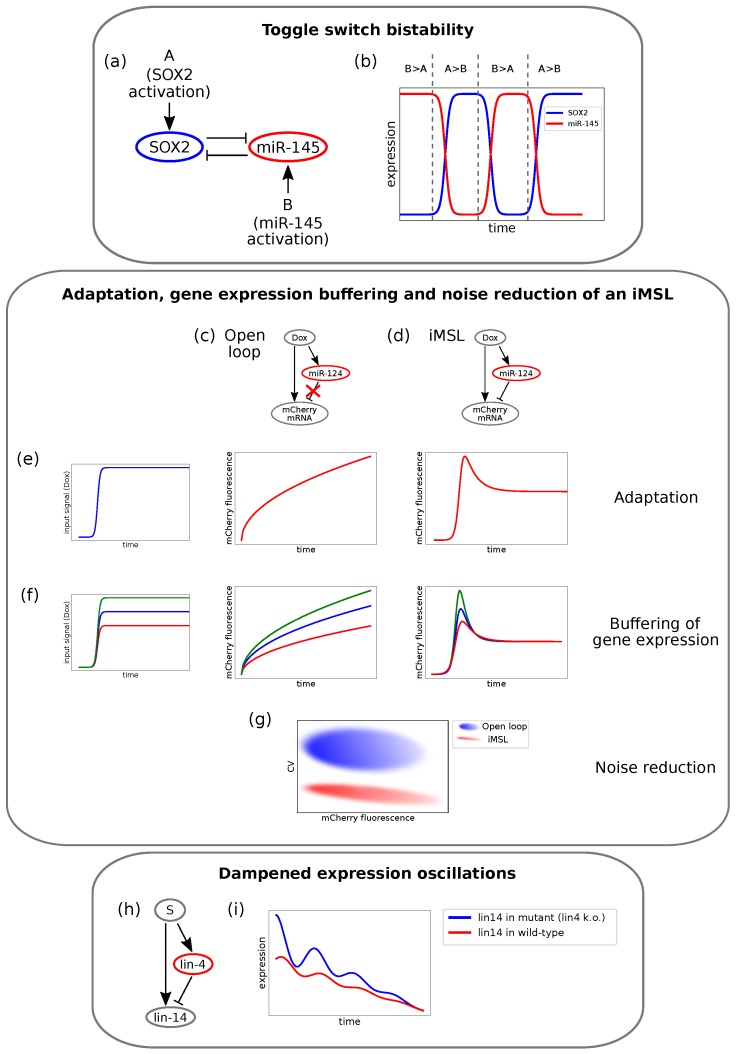
Examples of different experimental circuits showing properties theoretically predicted. (**a**) Example of the double negative feedback loop (DNFL) that displays bistability described in Fang et al. [100]. The circuit involves SOX2 and *miR-145*. (**b**) Bistable output of the DNFL. SOX2 temporal expression is depicted in blue and *miR-145* temporal expression in red. The relative activation strengths determine whether the system settles in the high-*miR-145* state or in the high-SOX2 state. Panels (**c**–**g**) report a schema of the results obtained by Strovas and colleagues [147] for an intronic miRNA mediated self-loop (iMSL). (**c**) The engineered open-loop. (**d**) The engineered iMSL. (**e**) Unlike the open-loop, the iMSL displays output adaptation. When a sudden input (left panel) is given to the system, the output fluorescence of the open-loop responds with an increasing trend (central panel), while the iMSL produces a pulse before reaching a lower steady-state level (right panel). (**f**) In the open-loop case (central panel), output levels depend on input signal intensities (left panel). On the contrary, the adaptive output level of the iMSL does not depend on the input size, and the iMSL buffers gene expression (right panel). (**g**) The iMSL displays noise reduction: for a given mean fluorescence value, the coefficient of variation (CV) is significantly lower in the iMSL case (in red) than in the open-loop (in blue). (**h**) The iFFL addressed by Kim and co-workers [120]. *Lin-4* and *lin-14* miRNAs are both transcribed by an upstream TF and *lin-4* post-transcriptionally targets *lin-14*. (**i**) *Lin-14* temporal expression in wild-type *C. elegans* (blue) and in mutant with *lin-4* transcription knock-out (red). *Lin-14* oscillation are dampened by *lin-4* pulsatile expression (adapted from [120]).

In order to gain deeper insight into the biological functions of the DNFL, it is convenient to address the case of an miRNA and a TF mutually inhibiting each other, with upstream signals that can activate them, as depicted in Figure 3a. The example considered here has been investigated by Fang and co-workers in the context of the glioblastoma multiform human cancer [100]. SOX2, a gene notably related to glioblastoma, forms a DNFL with *miR-145*. If *miR-145* transcription is more induced than that of SOX2, its repressive strength over SOX2 will overcome the opposite inhibitory arm, thus leading to a state where only the miRNA is expressed and SOX2 is silenced (Figure 3b, *miR-145*-high expression levels). On the contrary, if SOX2 transcription is favoured over *miR-145* transcription, the system collapses in the only-SOX2 state, where *miR-145* is silenced [100] (Figure 3b, SOX2-high expression levels). Therefore, the DNFL embodies a simple mechanism that allows achieving bistable expression of its components.

### 2.2. MicroRNA Mediated Feed-Forward Loops

The FFL is one of the most represented three node network motifs in transcriptional networks [1,101]. Given its role in a wide range of biological processes [102,103,104] and its well established association with diseases (ranging from cancer [17,105,106] to autoimmune diseases [107]), it is currently widely studied. Its general scheme is shown in Figure 2a, where the edges between the nodes represent the interactions between the molecules and may have either an activating or repressing function. The FFL is composed by a master regulator *A* that regulates the expression of a target *C* through two parallel paths: one direct and the other indirect. Through the latter, *A* directly regulates the expression of a second player, *B*, which in turn regulates *C* [2].

This type of circuit was firstly discovered in *Escherichia coli* [108] and then in several other organisms including yeast [1] and humans [101]. Later, different works showed an over-representation of FFLs mediated by microRNAs at a genome wide level [11,109,110,111], highlighting their importance in gene regulatory networks [112]. Specifically, in the case of mammalian genomes, these circuits play crucial roles in cell decision making, proliferation, development, and differentiation [6,102,103,104,113,114,115], as well as in DNA synthesis control and cell cycle regulation in both normal and cancer cells [111,116,117].

Different biological functions can emerge [42] depending on the interactions among the nodes of miRNA mediated FFLs, which are schematised in Figure 2a. FFLs are usually classified depending on the overall sign of their interactions, where the overall sign is calculated by multiplying the signs of each single path [2]. Based on this definition, the FFLs are classified as coherent (see Figure 2b–d) or incoherent (see Figure 2e,f) if the overall sign is either positive or negative, respectively. These two types of FFLs, as illustrated below, give rise to a wide range of features related to conferring robustness to the regulatory network in development and decision making.

#### 2.2.1. Coherent Feed-Forward Loops

Mutually exclusive spatial expression of miRNA and its target characterises coherent FFLs (cFFLs) [102]. The case shown in Figure 2c, where a TF activates the target and represses the miRNA, thus effectively reducing the miRNA mediated target repression, suggests a fail-safe control role of the circuit, since the miRNA and target cannot be co-expressed. This has been experimentally proven in the case where either the TF or the miRNA was the master regulator of the circuit [104] (Figure 2d). Evidence showing the lack of co-expression of the two molecular species has been observed during the development of *Drosophila* [121], as well as in mice [122] and epithelial tumours [105].

In the example of epithelial tumours, Kobayashi and colleagues found that two miRNA mediated cFFLs regulate the expression of genes specific to one of the two phenotypes that human epithelial tumour cells may present, called type 1 [105]. These cFFLs involve the miRNA *miR-199a*, a catalytic subunit of a complex that regulates gene transcription (Brm) and the transcription factor EGR1; see Figure 2g. One of the two cFFLs has the miRNA as the master regulator, which downregulates type 1 specific genes both directly and indirectly through the downregulation of Brm, which in turn activates the genes. The other cFFL has EGR1 as the master regulator targeting the same type 1 specific genes both directly and indirectly through the activation of *miR-199a*. The activity of both loops leads to a mutually exclusive expression of miRNA and both its targets, and the expression of the type 1 specific genes occurs in an “all-or-none” manner.

Interesting examples that underline the importance of miRNA mediated cFFLs in defining spatio-temporal boundaries during differentiation have been also found in other human cells, like neurons [103] and human fat cells [104]. In a recent work focused on the formation of new human fat cells, the authors identified a novel cFFL involved in adipocyte differentiation [104]. The network motif is characterised by an miRNA (*miR-27a/b-3p*) playing the role of the master regulator of a transcription factor (peroxisome proliferator activated receptor gamma (PPARG)) and of a second target gene, the secretory carrier membrane protein 3 (SCAMP3). SCAMP3 is then positively regulated by PPARG; see Figure 2h. Since the miRNA controls the expression of both target genes, it could either prevent the excess (or lack) of target transcripts [11,123] or fine tune the ratio of the expression of the target genes, the latter predicted through stochastic modelling by Riba and colleagues [117]. Kulyté and collaborators evidenced opposite effects of PPARG and SCAMP3 on adipogenesis and on the functions related to adipocytes [104]. PPARG positively regulates adipogenesis, while SCAMP3 is an endogenous limiter of adipogenesis driven by PPARG. In other words, SCAMP3 negatively regulates the process. The regulation of adipogenesis is then fine tuned by the enrolment of PPARG and SCAMP3 within the cFFL mediated by *miR-27a/b-3p*. Thus, the net effect of such a loop is to tune the ratio between PPARG and SCAMP3, which could be relevant for maintaining a balance between hypertrophic and hyperplastic expansion of adipose tissue in obesity.

In [117], Riba and coworkers, in support of their theoretical findings about cFFL, presented a cFFL involving the *miR-17* family as the master regulator, E2F1 as the transcription factor and RB1 as the target gene, positively regulated by E2F1; see Figure 2i. The motif is involved in the transition between the G0/G1 and S cell cycle phases, since E2F1 belongs to the family of genes that control such a transition. In phase G0, E2F1 and RB1 proteins are bound in complexes in almost all cells. In such a case, RB1 inhibits E2F1 functions and thus the cell cycle. On the contrary, in the presence of a stimulus that promotes cell division, the binding of E2F1 and RB1 is reduced, and the free-from-binding E2F1 proteins trigger the cell cycle. Thus, for a correct functioning of the transition between the two cell cycle phases, the relative concentration of the two genes must remain stable against fluctuations. The authors suggested that this is guaranteed by the miRNAs within the cFFL.

Another interesting example was shown in [52], where a cFFL limits the impact of noise in an oncogenic switch in response to an inflammatory signal [42]. The loop involves the transcription factor NF-κB, which directly activates a mediator of the inflammatory response, the cytokine interleukin-6 (IL6), and inhibits the miRNA *let-7*, which in turn represses IL6; see Figure 2j. Interestingly, the loop functions as an AND logic gate [3]: the activation of both the arms of the motif is necessary in order to reach the level of IL6 activity that allows cellular transformation. Such transformation is also aided by a positive feedback loop between NF-κB and IL6. The presence of *let-7* prevents the noise in the activity of NF-κB or IL6 from triggering the NF-κB/IL6 feedback loop.

The role of miRNAs in conferring stability to differentiation processes when embedded in cFFLs has been also highlighted in [115], where the authors investigated the role of *miR-7* in the differentiation of photoreceptor cells of *Drosophila*. The authors showed how two interlinked cFFLs with a TF as master regulator lead to a general expression stability. However, if the function of *miR-7* is compromised, this stability is lost, and the system can be perturbed by temperature variations.

Several theoretical works discussed the role played by cFFLs in managing fluctuations [112,117,124,125]. In [125], Duk and collaborators found that cFFLs do not show absolute adaptation in response to external signals, i.e., the target expression level does not return to its baseline level after a transient external signal, neither if the signal is small. The ability to adapt to different environmental conditions is an important feature for living organisms, especially in noisy environments. The absence of adaptability suggests that cFFLs may be present only in those systems where a substantial change of the target expression, even if induced by small changes in the external condition, is not deleterious.

#### 2.2.2. Incoherent Feed-Forward Loops

miRNAs may play a dual role in the presence of noise: on the one hand, they may generate and take advantage of fluctuations in order to perform defined tasks or to drive differentiation [44,119,126,127]; on the other hand, they can confer phenotypic robustness to gene expression by suppressing this variability [88,122,123,128,129,130]. In this framework, miRNA mediated circuits have been modelled as incoherent FFLs (iFFLs), as shown in Figure 2e,f. Stochastic modelling and numerical simulations were proven to be a powerful instrument to establish the role of miRNA in conferring robustness to these circuits by buffering gene expression fluctuations [6,48,128,131,132,133,134].

Furthermore, while cFFLs promote the avoidance of the spatial co-expression of miRNA and their targets, iFFLs can promote the temporal phase shift between molecular species. Let us consider an iFFL where a TF acting as the master regulator induces the expression of both the miRNA and the target (Figure 2e). Even if activated by the same TF as its own target, miRNA expression appears to be delayed as a consequence of its own biogenesis [11,102]. Yet, once expressed, the miRNA inhibits the target, therefore inducing the temporal phase shift mentioned above.

An example of an miRNA mediated iFFL with a TF as the master regulator was described in the work of O’Donnell and colleagues [118]. The circuit involves the proto-oncogene *c-MYC* that encodes a TF responsible for the regulation of cell growth, proliferation and apoptosis. The authors found that *c-MYC* directly controls the transcription of E2F1 and indirectly limits E2F1 translation through the activation of the expression of a cluster of miRNAs; see Figure 2k. In [130], He and collaborators investigated an iFFL similar to that of O’Donnell et al. involving again *MYC* as the master regulator, the *miR-17-92* cluster and E2F (E2F1 is a member of E2F family), and they showed that this type of loop effectively contributes to reducing gene expression fluctuations and therefore conferring to the miRNA the role of fine tuner of gene expression.

Analogously, Hilgers and collaborators [119] showed that the miRNAs *miR-263a/b* prevent programmed cell death of mechanosensory cells during fly retina development and confer robustness to the sensory organ specification (a role already proposed in [115] for the miRNA *miR-7* involved both in a cFFL and an iFFL). A scheme of this iFFL is shown in Figure 2l: *miR-263a* controls the expression of the pro-apoptotic gene *head involution defective* (*hid*) directly and indirectly through the RAS/MAPK pathway (composed in this case of *Ras85D* and *ERK*). With the sole direct path, the effect of the miRNA would be that of downregulating the expression of *hid* and thus its activity. However, this is mitigated by the indirect path through which *miR-263a* inhibits a *hid* repressor. The activity of the miRNA is thus to ensure robustness during apoptotic tissue pruning.

On the theoretical side, the work in [48] deeply studied the role of the iFFL in buffering gene expression fluctuations and compared it to other topologies by stochastic modelling and numerical simulations. Further works followed the one by Osella and colleagues [112,124,125,135,136]. More specifically, Duk and collaborators [124] confirmed the ability of the miRNA mediated iFFL to respond to a sudden change in the quantity of TF regulator with only a small deviation from the steady state. This allows highlighting the outstanding role of these regulatory motifs in those systems where changes in the target protein may cause serious consequences. However, this noise buffering property is evident only if the strength of the coupling between miRNA and target is tuned in a relatively small functional range. Outside this range, the circuit tends to either amplify fluctuations (strong coupling) or to produce a Poissonian statistics for the output variable (weak coupling) [137].

An important property of miRNA mediated iFFLs derived through mathematical modelling [125,138] is their ability to adapt to transient signals, as purely transcriptional iFFLs [139]. Bleris and collaborators [140] studied the adaptability to changes in DNA template abundance through the development of synthetic transcriptional and post-transcriptional iFFLs in human embryonic kidney cells. They found that in all cases, the gene product levels adapted to changes. In addition to this, the post-transcriptional form showed a better adaptive behaviour, higher absolute levels of expression and lower levels of intrinsic fluctuations when compared to the purely transcriptional case. These results, in agreement with theoretical expectations [112,124,125], support the endogenous role of this motif in gene dosage compensation. Thus, the implementation of miRNA mediated iFFLs in synthetic networks would improve their robustness. Yet, the ability of the miRNA mediated iFFL to control protein noise can be altered in the presence of bursting inputs, as shown through stochastic modelling and numerical simulations in [137].

Similarly to what has been discussed regarding FLs, iFFLs can produce oscillatory outcomes under peculiar conditions. An interesting example, carried out by Kim and collaborators [120] and reviewed in [141], consists of an iFFL involved in the development of *Caenorhabditis elegans*. The timing of its developmental stages is stressed by oscillatory expression patterns. It is known that *lin-4* oscillates according to these stages, whereas *lin-14* displays a seemingly constant negative gradient throughout development of the wild-type worm. By investigating the relationship between *lin-4* and *lin-14*, the authors found out that these two miRNAs are connected by an iFFL, as shown in Figure 2m and Figure 3h. By knocking down *lin-4* transcription, that is silencing the inhibitory arm of the iFFL, they showed that *lin-14* undergoes pulsatile dynamics as well. It is thus suggested that the pulsatile expression of *lin-4* dampens the expression oscillations of its target *lin-14*, thereby ensuring its temporal gradient. Temporal expression of *lin-14* in wild-type and in the *lin-4* lacking mutant is qualitatively represented in Figure 3i. Moreover, the dampening is shown to be optimal if *lin-4* and *lin-14* are transcribed together with in-phase pulses [120]. This could more generally represent a mechanism that serves to insulate key developmental players from upstream oscillatory or noisy signals. Several further examples can be brought forward to show that iFFLs are well suited to act as triggers for oscillatory systems and underline their importance in preventing the propagation of fluctuation in gene expression that might damage a developing organism [42,115].

iFFLs mediated by miRNAs have also been shown to detect gene expression fold changes [11,142]. Fold change detection is a feature related to Weber’s law: the response of a system depends on relative rather than absolute changes in the input signal with respect to a basal level. Mathematical modelling [143] showed that iFFLs display such a property for a wide range of biochemical parameters. Having a fold detection mechanism may allow identical responses to external signals despite the cell-to-cell variability in the basal levels of master TFs.

#### 2.2.3. Intronic microRNA Mediated Self-Loops

As mentioned above, the functions of many intronic miRNA appear to be correlated with those of the genes encoding the miRNAs themselves [144]. Using measures based on both annotation and experimental data, Lutter et al. suggested that many miRNAs link host and target gene activity in an either synergistic or antagonistic manner [145]. Here, we will focus on a minimal interaction circuit that sees intronic miRNAs as direct repressors of their host gene, that is the intronic miRNA mediated self-loop (iMSL), shown in Figure 2n. The iMSLs, which can be viewed as a compact version of an iFFL, can be represented by a master TF that regulates a single genomic locus encoding both the host gene and the miRNA, with the host gene in turn targeted by the miRNA [13]. Bioinformatic studies have found that iMSLs are significantly enriched in the human regulatory network [146], with approximately 20% of intronic miRNAs predicted to target their host gene [144]. This suggests that iMSLs can perform specific biological functions, potentially not achievable by other circuit topologies.

The question of the reason why intronic miRNA mediated gene autoregulation would be more suitable to fulfil certain tasks has been addressed both theoretically and experimentally [13,135,147]. Bosia and co-workers carried out a controlled mathematical comparison between the iMSL and other circuit topologies in order to highlight the iMSL’s specificities in implementing particular functions. The results indicated that the iMSL can accelerate the synthesis of the host gene protein in response to an input signal and can delay its knockdown when the input signal drops. As other circuit topologies, it can efficiently buffer fluctuations of the upstream signal. In addition, the iMSL is able to implement complex functions such as a host gene expression obeying Weber’s law, that is the response only depends on the input’s fold change and not on the input’s absolute value [13].

Strovas and colleagues [147] engineered a synthetic iMSL circuit composed of a doxycycline inducible fluorescent reporter self-regulated by its intronic miRNA *miR-124* (Figure 3d). As a control, the authors engineered an analogous loop lacking the inhibitory arm (open-loop, Figure 3c). By integrating these circuit into the cells’ genome and then measuring the fluorescent reporter amounts, they found that the iMSL showed adaptation (i.e., a transient protein expression pulse before returning to a lower steady-state level in response to a step-like increase in transcription rate), while the open-loop did not (Figure 3e). Moreover, the steady-state protein levels for the iMSL were independent of the size of the stimulus, thereby buffering protein production against changes in transcription (Figure 3f) and reducing cell-to-cell expression variability (Figure 3g). Yet, to this concern, a newly published study highlighted the dependence of the iMSL’s noise buffering efficiency on the time scale of fluctuations [135].

A series of endogenous iMSLs have also been experimentally uncovered in small scale studies [148,149,150,151,152,153,154]. Chuang et al. [155] found *miR-93-3p* and MCM7 to be oppositely expressed in leiomyomas. Since the MCM7 gene encodes a protein that plays an important role in cell cycle, *miR-93-3p* may regulate replication and cell cycle progression through an iMSL. Another iMSL involving *miR-26b* was found to control neuronal differentiation by post-transcriptional repression of its host gene, CTDSP2 [149]. However, the experimental validations of this kind of circuit are still in their infancy, as only a few examples of iMSLs have been confirmed to date [156].

Summarising, the iMSL appears in general to be another useful way of maintaining cellular homoeostasis and conferring robustness to gene expression, coherently with the emerging idea of miRNAs as fine tuners of biological processes.

## 3. MicroRNAs in Synthetic Circuits and Therapeutic Perspectives

In the previous sections, we summarised and discussed the main properties of miRNA mediated gene regulatory networks, with particular attention to network motifs. Up to this point, the properties of endogenous circuits have been discussed. However, the ability of engineering synthetic regulatory networks, as well as integrating them in cells through plasmids or viral vectors gives the unique opportunity of investigating complex networks’ behaviours under highly controlled conditions [157]. Besides representing a powerful experimental setup to test the predictability of theoretical results, these techniques, globally referred to as synthetic biology, give a chance to convey the first milestone results for therapeutic purposes.

Synthetic biology is based on a strongly interdisciplinary approach called *cybergenetics* [158], where the concepts of cybernetics are integrated with control theory, biological engineering and mathematical modelling in order to design synthetic circuits able to tune gene expression and thereby control cell functions [158,159]. The design of these circuitries able to sense multiple biological inputs, process complex information and finally give physiologically active responses is a long-standing challenge [160]. In particular, the reprogramming of genomic activities in mammalian cells could provide therapeutic strategies for a variety of diseases [161]. For instance, regulatory networks built to detect and selectively react to certain cellular conditions could exert anti-cancer actions [162].

A vast amount of studies have focused on the design of regulatory motifs controlled by transcriptional signals [160]. After purely TF mediated synthetic circuits, recent proposals rely on RNA interference (RNAi) logic; that is, the circuit’s outcome depends on the interference of some RNA molecule with protein production. Specifically, engineered miRNAs are now widely used as circuit modules in order to exploit their well discussed regulatory features [163]. Generally, networks based on RNAi are constructed by coupling the binding of an input ligand to an RNA aptamer with the biogenesis of the RNAi [164]. In fact, many studies used a ligand that binds to the aptamer and thus causes conformational changes that inhibit miRNA biogenesis [165], thus affecting the production of an miRNA targeted protein [165].

Leisner et al. [160] proposed a general method for implementing transcriptional regulatory programs with RNAi circuits in mammalian cells by transfection. Their model has multiple TF inputs and a fluorescent protein output, and it is composed of different modules. In one module, each TF regulates a sensory miRNA gene. The mature miRNA converges on its mRNA target, which encodes an output protein tagged with a fluorescent dye that enables its quantification. By calibrating the features of constitutive regulator TF expressing vectors, miRNA expressing vectors and reporter plasmids encoding targets, the circuit’s response can be tuned. This approach is presented as a basic starting point for more complex synthetic circuits that make use of RNAs [160].

**Figure 4:**
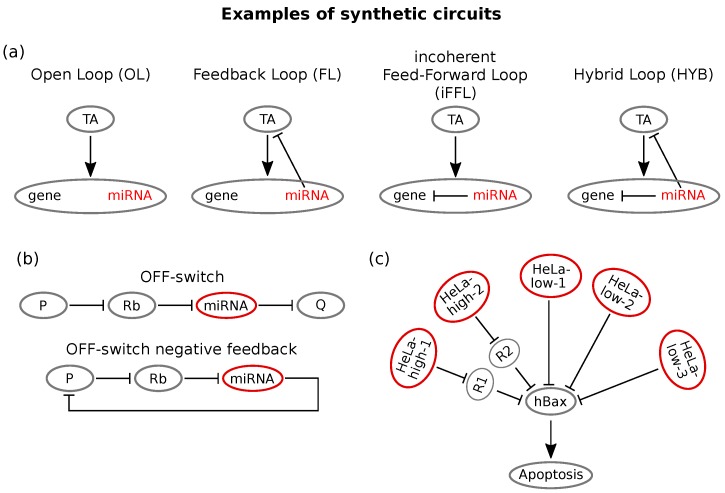
Examples of synthetic circuits. (**a**) Schemes of the four miRNA based controllers engineered in Lillacci et al. [158]. All the implemented circuits involve the same upstream regulator (tetracycline-controller transactivator TA), which targets a gene with intronic miRNA. In the open-loop (OL), the miRNA cannot bind the target. In the feedback loop (FL), TA is provided with miRNA binding sites; thus, a feedback is formed between the two species. In the incoherent feed-forward loop (iFFL), the gene is targeted by its intronic miRNA, thus forming an intronic miRNA mediated self-loop. In the hybrid loop (HYB), both the upstream regulator TA and the gene are targeted by the miRNA; thus, a hybrid-type loop is formed. (**b**) Scheme of the OFF switch implemented by Bloom et al. [165]. The ligand, an input protein P, inhibits the ribozyme (Rb), thereby preventing its cleavage action on the miRNA. Thus, miRNA biogenesis occurs, and the target gene G is repressed. In the following phase, the OFF switch is shaped in such a way that the target coincides with the input protein itself, thus forming a controlled feedback loop that exhibits input level adaptation. (**c**) The identified miRNAs (two HeLa-high and three HeLa-low markers) target the same apoptotic gene, hBax. Adapted from [162].

An example of great interest for the purposes of this review is the work of Lillacci and collaborators [158], who reported new controllers of gene expression in mammalian cells containing post-transcriptional feedback and feed-forward regulation mediated by miRNAs. The authors designed and transfected in mammalian cells four controllers based on RNAi logic. The four implemented circuits were: (i) an open-loop (OL), (ii) a feedback loop (FL), (iii) an incoherent feed-forward loop (iFFL) and (iv) an hybrid of feedback and incoherent feed-forward loops (HYB). All of these circuits were implemented using plasmids with the same genes, and the only difference among the four was due to the regulatory relationships among them. The plasmids were composed by (i) a constitutively expressed yellow fluorescent protein (mCitrine) that represents a proxy for the amount of plasmid uptaken by the cell, (ii) a blue fluorescent synthetic controller (tetracycline-controller transactivator tTA-Advanced, called TA, fused to Cerulean) that induces the expression of both; a (iii) red-fluorescence protein of interest (DsRed) and (iv) an intronically encoded miRNA (*miR-FF4*). In order to implement different circuitry logics, the 3′-UTR regions of both TA and DsRed were engineered to contain or not binding sites for *miR-FF4*. The activity of TA over its downstream gene was externally tuned through doxycycline (DOX), which inhibits TA activity. Quantification of the expression levels has been performed through flow cytometry. Figure 4a reports the four loops.

The open-loop represents the standard inducible gene expression system and is engineered without *miR-FF4* binding sites: TA therefore induces the expression of both the miRNA and the target. In the feedback loop instead, TA is provided with miRNA binding sites, therefore defining a feedback loop between the miRNA and its activator. The feed-forward loop is instead implemented via an intronic miRNA self-loop: *miR-FF4* binding sites are on the 3′-UTR region of the DsRed; thus, both miRNA and its target are activated, and the miRNA represses the target. Finally, the hybrid loop (HYB) is the union of the last two loops: miRNA binding sites are present on both TA and DsRed.

Through such controllers, the main properties of the different loops discussed in the previous sections have been recovered. In particular, the authors showed that (i) the negative FL was able to confer robustness to TA dosage variability and improved output protein yield, (ii) the iFFL could tune the output expression and confer robustness to variability due to plasmid uptaking and (iii) the hybrid circuit showed ability in tuning the output, conferring robustness to plasmid uptaking variability, and showed adaptation to gene dosage. Moreover, the authors integrated such circuits in biologically interesting systems in order to improve their performances. For instance, the transfection of the FL into Chinese hamster ovary cells, usually implied in protein manufacturing, increases product levels by eliminating the burden effect induced by TA. Burdening represents indeed one of the main drawbacks in bioproduction affecting cell physiology: several bioengineering efforts have been made in order to limit this effect [166,167]. Moreover, the advantages of iFFL and HYB circuits have been observed by their transient transfection in human induced pluripotent stem cells. In the control situation, high levels of transgene expression were toxic to the cell line, and the presence of either iFFL or HYB allowed mitigating the toxic effect by regulating gene expression.

Inspired by the property of some miRNA mediated network motifs of preserving intracellular protein levels, cellular homoeostasis maintaining RNAi networks have been engineered [165]. A study by Bleris et al. [140] showed that the iFFL implements gene expression adaptation in mammalian cells. Specifically, the authors synthesised and tested FFLs based either on transcriptional or post-transcriptional regulation and observed that the output recovered adaptive levels in response to template abundance variation. Another interesting homoeostasis controlling system has been developed by Bloom and colleagues [165]. It consisted of a synthetic FL that combines a ligand responsive ribozyme switch and synthetic miRNA regulators. While most of RNAi devices function as ON-switches, that is the output protein’s expression increases with ligand concentration, they developed a circuit that conversely increases silencing of the product by an miRNA in the presence of a ligand, thereby realising an RNAi based OFF-switch. This was possible by using a ribozyme (Rb) whose presence ensures pri-miRNA formation and consequently silences the target gene. This OFF-switch was then used to target the ligand protein itself in an miRNA-controlled negative feedback that maintains constant protein levels in response to increases in the transcription rate [165], as shown in Figure 4b. This type of circuit can be a powerful way of stabilising cellular phenotypes, thus having useful therapeutic implications [165].

Of course, the well known simple network motifs can be combined in order to obtain better performing synthetic circuits [168]. Reeves and co-workers analysed the effects of combining different synthetic motifs on output adaptation and robustness: they combined an FFL with a negative feedback and showed that this network showed more robust dynamical adaptation properties than simple FL or FFL systems [168].

### MicroRNAs as Classifiers

A network’s ability to implement gene expression conditions can also be used for recognising cancer cells and triggering their apoptosis [164]. This idea lies behind the popular recent field of cell classifiers in synthetic biology [157,169]. A classifier is a regulatory network that detects sensory information from multiple molecular markers to determine if a cell is in a specific state and to produce consequently a biologically active protein output [162]. A major challenge for cancer therapy is to eliminate cancer cells without damaging the surrounding healthy ones [161]. In this view, cell classifiers appear to be great candidates to sense pathological cell conditions and to generate targeted therapeutic responses [161].

MiRNA responsive circuits are particularly useful for these purposes, as miRNA expression can be seen as a signature of the cell’s condition and identity [159]. In this view, Xie and co-workers built a synthetic miRNA mediated circuit capable of distinguishing cervical cancer cells (HeLa cells) from other cell types. This circuit senses the expression intensities of a customisable series of endogenous miRNAs and activates a cellular response if these intensities match a pre-established profile [162]. More precisely, it can sense the expression of six different miRNAs and compare it to a previously constructed reference profile that is supposed to identify HeLa cells [162]. Depending on the output, it can then trigger the expression of an apoptosis inducing protein, hBax. A few miRNAs were identified as HeLa-low or HeLa-high markers. Then, the circuit’s response function was modelled as a combination of logic AND/AND NOT operations on all miRNA inputs. This computation can be built in a network by choosing a homogeneous pool of miRNA targets (hBax expressing gene, Figure 4c) and implementing two feedforward loops with a threshold-like output expression. The constructed circuit performed as desired, the outcome being significantly higher in HeLa cells compared with the other cell lines used. Moreover, it selectively killed HeLa cells with respect to other cells [161].

Besides the usefulness of miRNA sensing in order to select apoptotic cells, Hirosawa et al. showed that endogenous miRNA profiles can be used by a regulatory switch for controlling genome editing [170]. A synthetic network can trigger effective biological programs by detecting complex intracellular signals. Nevertheless, therapeutic applications require the overcoming of challenges such as efficient in vivo DNA delivery to cells [162]. Moreover, as DNA delivery can lead to random genomic integration, RNA delivered circuits are preferable for medical purposes in terms of safety [159]. A recently published work [159] used an all-RNA system that provides an alternative to DNA delivery. A multiple miRNA sensing circuit for logic computation in mammalian cells was constructed using an RNA-only delivery approach. This kind of RNA based logic circuit with RNA binding proteins tunes the output’s production with the aim of selectively controlling cell death pathways. Five logic gates (AND, OR, NAND, NOR, XOR) were created and validated by measuring the output’s net fold-change, and all circuits displayed statistically significant performance [159]. Specifically, a two-input AND circuit involving an apoptotic gene led to precise elimination of target cells.

More generally, circuit topology can be optimally shaped in order to generate the best candidate circuits for precise cell targeting and avoid the extensive use of empirical trials. This issue has been addressed by Mohammadi et al. in a recent work [171] by using again miRNA expression datasets as phenotype markers. In this study, a computational method was presented for designing experimentally feasible miRNA mediated circuits that can distinguish cells according to various actual classification problems [171]. A unique circuit specification comprises the set of inputs, the network topology, and a set of biochemical parameters such as rate constants and initial conditions that determine its dynamics [171]. Obviously, optimisation is burdened by the combinatorial explosion of the number of possible circuits. A two step approach can be used to solve this issue: first, an optimised set of biochemical parameters is derived over a set of different topologies; second, the circuit topology is optimised for each task using the globally inferred parameters obtained in the first step [171]. In sum, this work provides a method that identifies a single circuit with optimal classification performance, that is producing the highest output levels in target cells with respect to all other cells [171].

Yet, the optimisation of single circuit classifiers often suggests the design of highly complex structures that are difficult to build in the real world [157]. A newly published study by Nowicka and colleagues made use of the so-called distributed classifiers (DC), that is sets of small and simple network motifs that function in an integrated manner and produce a collective response according to a threshold function [157]. Specifically, an initial set of simple networks is trained using a machine learning algorithm, and as a consequence, low-performance circuits are withdrawn. By using real cancer data, it was shown that such a set of simple classifiers performs better in terms of classification and is more robust with respect to a single complex circuit, as the integration of individual outputs compensates for single circuit errors. This suggests that DCs may also manage noise in the input data better than single network classifiers [157].

More recently, the achievements of synthetic circuits have gone beyond the simple cell type recognition thanks to a new work by Endo and colleagues [172], which showed that their mRNA based circuits are not only capable of discriminating human cells, but they can also track their change when they differentiate. Thus, their work opened the possibility to make decisions also based on dynamical information on the intracellular state.

Cell dynamics tracking of course opens questions about how to reprogram differentiated cells to pluripotent cells [173] or how to control stem cell differentiation precisely [174]. Yet, one of the greatest challenges for clinical applications is that of ensuring that engineered cells do not present side effects to humans [161]. To lower this risk, host cells should be replaced by primary cells or stem cells isolated directly from the patient. Thus, induced pluripotent stem cells may pave the way to personalised medicine by allowing the generation of patient specific cells and tissues that do not have to be derived from embryos [173].

As discussed, miRNA mediated synthetic networks open a wide range of useful biomedical possibilities. Future perspectives of miRNA mediated synthetic motifs involve the development of circuits with high biocomputing capacity, able to integrate a large number of diverse inputs in order to provide always more sophisticated biological responses.

## 4. Conclusions

The network motifs discussed so far were generally considered as single blocks composed by a small number of interacting units. Despite their simplicity, we showed how they are able to give rise to complex biological functions linked to noise processing. However, by enlarging the picture, they represent single building blocks of the bigger and more complex network that regulates gene expression in eukaryotes.

As already mentioned in some of the examples reported in the previous sections [52,115,130], the regulatory output and thus the role of the single players is often the result of the interconnection of some of the elementary circuits, like the combination of a feed-forward loop with a feedback loop [52,130]. For instance, in [130], an interplay between miRNA mediated feedback and feed-forward loops that share some of their components may provide the miRNA with the dual role of oncogene and tumour suppressor. In addition to this, a specific biological feature (see for example the ability of suppressing noise) may be achieved through different pathways, suggesting, from a mathematical point of view, the non-uniqueness of the solution [11,112].

The NF-κB regulatory network displays a striking example of this behaviour. As discussed, NF-κB is involved in a series of interconnected miRNA mediated loops that regulate inflammatory response. However, the regulatory relationships between miRNAs and NF-κB go well beyond the few loops shown so far. Markopoulos et al. [175] reviewed the connections of NF-κB with several miRNAs that modulate inflammation in cancer and discussed the role of each of these connections. For instance, an NF-κB/*miR-155* network appears to calibrate the intensity and duration of inflammation, whereas the NF-κB/*miR-21* loop displays an amplification behaviour that can lead from inflammation to cancer [175]. Furthermore, Rokavec and colleagues reviewed the roles of different NF-κB involving circuits in linking inflammatory conditions to cancer initiation and progression [176]. Since we cannot entirely report the described NF-κB network, an idea of its importance and ubiquity can be caught from the impressive number of NF-κB/miRNA interactions: genomic data provide a list of 162 miRNAs regulating NF-κB and, vice versa, 40 miRNAs targeted by NF-κB [175]. In sum, it is likely that the net outcome of NF-κB’s dense network can either favour cancer development or limit it. Moreover, some miRNAs that are regulated by NF-κB in turn regulate the already mentioned p53 involved in breast cancer development and vice versa [177]. Thus, the NF-κB and the p53 interactomes are connected as well, and together, they may determine the neoplastic transformation of a cell or oppositely tumour suppression [177].

By looking more closely at the p53 interactions with miRNAs, again, a number of p53 regulated and p53 regulating miRNAs have been identified. These miRNAs appear to be involved in cell cycle regulation, stemness regulation and cell survival, amongst others. Focusing on their cancer related roles, a part of these miRNAs acts as tumour suppressors, while some of them play oncogenic roles in the intricate p53/miRNA network [178]. An outstanding example among the p53 related miRNAs is represented by the *miR-34* family members, which have been found as the prevailing p53 induced miRNAs, as reviewed in [179]. Coherently with the well known relation of p53 with several types of tumours, this miRNA family has been implicated in diverse cancer related functions, such as proliferation, apoptosis, EMT, migration, and metastasis, as summarized by Rokavec and colleagues [179] and Yamakuchi et al. [180].

Furthermore, the epithelial-to-mesenchymal transition has been shown to be also connected to NF-κB. Indeed, the inflammatory signals that activate NF-κB also feed back on EMT inducing TFs, such as the already mentioned SNAIL and ZEB. For a review, see [181]. The NF-κB and p53 interactomes only give an insight into the importance of considering the ensemble of simple interactions as a whole. In sum, the functions of single motifs must be taken collectively into account in order to predict global biological responses, especially for therapeutic aims.

By zooming out from the simple network motifs, these may be part of a bigger and more complex regulatory network composed by the miRNAs and all their target genes. Indeed, the miRNA–target interaction has a combinatorial nature: one miRNA can regulate several targets, and these in turn can be regulated by several miRNAs. Such a feature, coupled with the molecular sequestration mechanism of miRNA-target binding, may induce cross-talks among the targets that compete to bind to the same miRNA pool, giving rise to a layer of post-transcriptional cross-regulation recently called the ceRNA (competing endogenous RNA) effect [22]. The number of binding sites in ceRNA candidates is inversely related to the effective concentration of free targeting miRNAs, making the shared miRNA binding sites cross-talk hubs of gene interactions. In light of this, the deletion of shared miRNAs by ceRNAs and the competition for binding represents an indirect mechanism for cross-regulation and interaction among RNA species [11].

Some examples in the literature show functional effects of ceRNAs in contexts like cancer [19,26], cell differentiation [182] and self-renewal of embryonic stem cells [183]. However, up to now, the broader characterisation of ceRNA properties has been performed under a theoretical point of view both through stochastic modelling and in silico simulations [20,21,45,184,185], as reviewed in [186]. Quantitative experiments that allow testing theoretical predictions are much more recent and still limited, and the best way to address such issues experimentally seems the use of synthetic biology. The study of engineered genetic circuits [187], in particular through the use of bidirectional reporters [18,188,189], allows investigating such complex regulation under controlled conditions in an isolated biological setting. A similar characterisation in those systems with evidence of functional ceRNAs [26,182,183] would be interesting, but it is still lacking.

## Abbreviations

MDPI	Multidisciplinary Digital Publishing Institute
DOAJ	Directory of open access journals
TLA	Three letter acronym
LD	linear dichroism
